# Rationale and design of the randomised controlled cross-over trial: Cardiovascular effects of empaglifozin in diabetes mellitus

**DOI:** 10.1177/14791641211021585

**Published:** 2021-06-28

**Authors:** Sharmaine Thirunavukarasu, Louise AE Brown, Amrit Chowdhary, Nicholas Jex, Peter Swoboda, John P Greenwood, Sven Plein, Eylem Levelt

**Affiliations:** Multidisciplinary Cardiovascular Research Centre and Biomedical Imaging Science Department, Leeds Institute of Cardiovascular and Metabolic Medicine, University of Leeds, Leeds, UK

**Keywords:** Empaglifozin, cardiovascular magnetic resonance imaging, Type 2 diabetes, continuous glucose monitoring

## Abstract

**Background:**

Type 2 diabetes (T2D) is associated with an increased risk of cardiovascular (CV) disease. In patients with T2D and established CV disease, selective inhibitors of sodium–glucose cotransporter 2 (SGLT2) have been shown to decrease CV and all-cause mortality, and heart failure (HF) admissions. Utilising CV magnetic resonance imaging (CMR) and continuous glucose monitoring (CGM) by FreeStyle Libre Pro Sensor, we aim to explore the mechanisms of action which give Empagliflozin, an SGLT2 inhibitor, its beneficial CV effects and compare these to the effects of dipeptidyl peptidase-4 inhibitor Sitagliptin.

**Methods:**

This is a single centre, open-label, cross-over trial conducted at the Leeds Teaching Hospitals NHS Trust. Participants are randomised for the order of treatment and receive 3 months therapy with Empagliflozin, and 3 months therapy with Sitagliptin sequentially. Twenty-eight eligible T2D patients with established ischaemic heart disease will be recruited. Patients undergo serial CMR scans on three visits.

**Discussion:**

The primary outcome measure is the myocardial perfusion reserve in remote myocardium. We hypothesise that Empaglifozin treatment is associated with improvements in myocardial blood flow and reductions in myocardial interstitial fibrosis, independent of CGM measured glycemic control in patients with T2D and established CV disease.

**Trial registration:**

This study has full research ethics committee approval (REC: 18/YH/0190) and data collection is anticipated to finish in December 2021. This study was retrospectively registered at https://doi.org/10.1186/ISRCTN82391603 and monitored by the University of Leeds. The study results will be submitted for publication within 6 months of completion.

## Background

**Figure 1.**
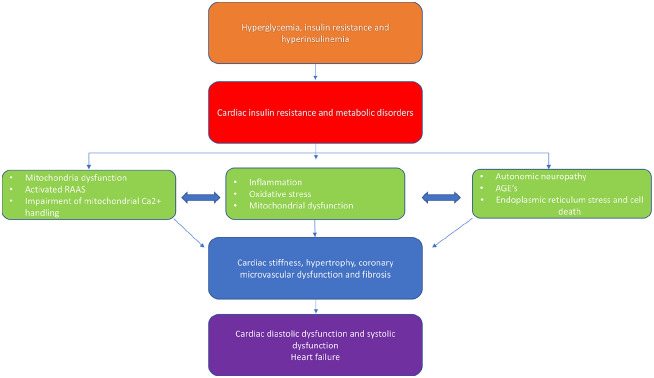
Potential molecular and macroscopic pathophysiological mechanisms leading to cardiac dysfunction in type 2 diabetes. In diabetes, insulin fails to suppress hormone sensitive lipase secretion in adipose tissue and very low-density lipoprotein secretion in the liver, leading to high circulating fatty acids.^29,30^ When fatty acid availability exceeds fatty acid oxidation rates, intramyocardial lipids accumulate. The subsequent lipotoxicity plays a role in the development of contractile dysfunction and observed in the diabetic heart.^31^ Moreover, inhibition of pyruvate dehydrogenase (due to the effects of pyruvate dehydrogenase kinase four induction and fatty acid derived acetyl-CoA) limits pyruvate oxidation.^32^ The dissociation of glycolysis and pyruvate oxidation in the diabetic heart results in the accumulation of glycolytic intermediates and glucotoxicity.^39^ Hyperglycemia, insulin resistance and hyperinsulinemia induce cardiac insulin resistance and metabolic disorders leading to mitochondrial dysfunction, oxidative stress, advanced glycation end products (AGEs), impairment of mitochondrial Ca^2+^ handling, inflammation, activation of renin–angiotensin–aldosterone system (RAAS), autonomic neuropathy, apoptosis and endothelial dysfunction. These pathophysiological abnormalities promote cardiac stiffness, hypertrophy, coronary microvascular dysfunction and fibrosis, resulting in cardiac diastolic dysfunction, systolic dysfunction and heart failure.^33^ Figure adapted from Jia et al.^33^

Cardiovascular (CV) disease is the leading cause of morbidity and mortality in patients with type 2 diabetes (T2D).^1^ The cardiovascular effects of T2D are characterised by multiple interconnected mechanisms and involve the cardiomyocyte, fibroblast and endothelial cell (Figure 1). A significant breakthrough in contemporary cardiology was the finding that sodium–glucose-cotransporter-2 (SGLT2) inhibitors are associated with a lower risk of heart failure (HF) hospitalisation in patients with or at high risk of CV disease.^2^ A selective inhibitor of SGLT2, Empagliflozin, reduces rates of hyperglycaemia in T2D patients by decreasing renal glucose reabsorption, thereby increasing urinary glucose excretion.^3^ In addition, SGLT2 inhibition causes a modest rapid reduction in weight, haemoconcentration and reduced blood pressure, consistent with a diuretic effect (2,3).

In the EMPAREG OUTCOME Trial, Empagliflozin reduced cardiovascular death and hospitalisation for heart failure (HF) by 38% and 35%, respectively, with an almost immediate beneficial effect despite only a modest difference in glycaemic control, comparing two study arms over 94 weeks.^2^ The reductions in CV death were not accounted for by the reductions in atherothrombotic outcomes, as the rates of myocardial infarction and stroke remained unchanged with therapy.^2^ The proposed theory that HF is the outcome most sensitive to SGLT2 inhibition was confirmed in the Canagliflozin Cardiovascular Assessment Study (CANVAS) Program and Dapagliflozin DECLARE–TIMI 58 trials.^4,5^ More recently, the EMPEROR trial showed that SGLT2 inhibition reduces the risk of hospitalisation for HF in patients regardless of the presence or absence of diabetes.^6^

The mechanisms by which SGLT2 inhibitors cause the reduction in HF admissions and cardiovascular mortality are as yet unknown, however recently suggested theories include their impact on coronary microvascular function and pleiotropic anti-fibrotic effects.^7^ Extensive evidence has documented the presence of coronary microvascular dysfunction^8–12^ and myocardial fibrosis^13–15^ in patients with T2D. Coronary microvascular dysfunction in diabetes is likely to be a multifactorial phenomenon, related to changes in perivascular and interstitial fibrosis,^16^ myocardial hypertrophy,^17^ reduced capillary density and autonomic neuropathy.^18^ Regarding the important drivers of interstitial fibrosis process in T2D, these include collagen cross-linking via accumulation of advanced glycation end-products, activation of inflammatory cytokines and potentiation of neurohormonal cascades such as upregulation of the renin–angiotensin–aldosterone system (RAAS).^19^ Importantly, both coronary microvascular dysfunction and interstitial fibrosis are both early precursors of cardiovascular events and were both shown to be associated with a 2–3 fold increased risk of annual major adverse event rate that includes cardiovascular mortality, nonfatal myocardial infarction, nonfatal stroke and congestive HF even among patients without epicardial coronary artery stenosis.^20,21^ The purpose of this study is to evaluate effects of SGLT2 inhibitor Empagliflozin on myocardial blood flow and myocardial interstitial fibrosis in patients with T2D.

### Cardiovascular magnetic resonance imaging in type 2 diabetes

Cardiovascular magnetic resonance imaging (CMR) is the reference standard for assessment of cardiac volumes, mass and function and also allows assessment of ischaemia and fibrosis.^22^ Using CMR, patients with T2D have been extensively phenotyped with a nuanced description of disease burden.^14,23,24^ These studies have identified predictors of adverse CV events including distinct ventricular morphology,^10,25^ reduced aortic distensibility,^14,23,24^ impaired strain,^12,14,24,26–28^ elevated myocardial extra cellular volume fraction indicating diffuse cardiac fibrosis^14^ and reduced myocardial perfusion^10,11^ in patients with T2D.

To our knowledge, the effects of Empagliflozin on myocardial perfusion, function, aortic distensibility and interstitial fibrosis has never been compared to other glucose lowering therapies. Similarly, antifibrotic properties of Empagliflozin compared with other agents remain to be shown in clinical studies of T2D. Optimising glycaemic control alone has failed to improve short to medium term mortality but recent trials of SGLT2 inhibitors provide new perspectives. Despite similar reductions in levels of glycaemia, only SGLT2 inhibitors and glucagon like peptide-1 receptor agonists improve CV outcomes, suggesting pleiotropic effects, potentially on myocardial fibrosis, aortic stiffness and myocardial perfusion effects.

### Continuous glucose monitoring- FreeStyle Libre Pro Sensor

Continuous glucose monitoring (CGM) provides the advantage of measuring interstitial glucose every 5–15 min, thus providing a comprehensive 24-h glycemic profile, with better assessment of nocturnal and/or asymptomatic hypoglycaemia and pattern recognition after each treatment intervention.^20,21,29–32^ Patients with T2D may experience hypoglycaemic events or increased glucose variability, both of which are linked to atherothrombotic vascular pathology and adverse clinical outcomes.^3,21,22,29,33^

Glycemic variability is considered an important glycemic target, together with glycosylated haemoglobin (HbA1c), to reduce the risk of diabetes complications. As this study aims to assess the cardiovascular beneficial effects of Empagliflozin independently of the drug’s action on glucose levels, the FreeStyle Libre Pro Sensor will be used for reliable continuous monitoring of glucose levels.

## Methods/study design

### Participants, interventions and outcomes

The CEED trial (Cardiovascular Effects of Empaglifozin in Diabetes Mellitus) is a single centre, open-label, randomised, cross-over study with blinded analysis of CMR data. The study has been registered at https://doi.org/10.1186/ISRCTN82391603

### Aims of the study

The study is designed to determine the effects of Empagliflozin on cardiac structural and functional changes, perfusion and aortic distensibility in a population of patients with similar characteristics to those studied in recent clinical trials^2^ and compare them with the effects of Sitagliptin. Patients will be administered the two drugs sequentially. The study will involve physiological assessment including CMR imaging of biventricular volumes, function, quantitative adenosine stress perfusion imaging, myocardial extracellular fibrosis and aortic distensibility. Additionally, the impact of Empagliflozin and Sitagliptin on plasma glucose variability will also be assessed by FreeStyle Libre Pro Sensor.^34^

### Original hypotheses

Cardioprotective mechanisms of Empagliflozin include improvement of myocardial perfusion and aortic distensibility, and reduction in cardiac fibrosis in patients with T2D and established cardiovascular disease. These beneficial effects are independent of Empagliflozin’s action on glucose levels.

### Justification of the hypothesised beneficial cardiovascular mechanisms and the mechanistic study

#### SGLT2 inhibitors improve blood pressure and vascular function

Significant BP reductions are detected after only 2 days of SGLT2 inhibition in T2D patients and SGLT2 inhibition almost immediately improves systemic endothelial function and arterial stiffness.^35^

#### SGLT2 inhibitors have cardiac antifibrotic properties

In the experimental work using human cardiac fibroblasts, Empagliflozin had direct effect on human cardiac fibroblast phenotype and function, particularly by attenuation of myofibroblast activity and cell-mediated collagen remodelling.^36^

### Justification of the comparator glucose-lowering drug Sitagliptin

To test the CV beneficial effects of SGLTi independently of the actions of the drug on glycemic control, it is important to compare SGLT2i treatment with a specific and clinically relevant treatment strategy, such as, dipeptidyl peptidase-4 (DPP-4) inhibitor therapy. The DPP-4 inhibitors belong to a class of widely used glucose lowering treatment for T2D, which have been shown to be associated with CV safety in large clinical trials.^37,38^ As such, DPP-4 inhibitors are a well-suited comparator for examining the effectiveness of SGLT-2 inhibitors.

### Patient and public involvement

We have worked closely with patients when developing this research and their voiced unmet needs have been pivotal in forming the research questions and study design. We have presented the study hypotheses and the proposed investigation methods, as well as their potential burden on the participants, to the Leeds Patient and Public Involvement (PPI) group and to the Leeds Diabetes UK Volunteer Group, and have incorporated the volunteers’ suggestions into the study protocol. In addition, Leeds PPI group with members of the public have reviewed all study documentation prior to application for ethics committee approval for the study protocol.

A Study Steering Committee, providing overall supervision of the study progress, adherence to protocol, participant safety and consideration of new information, includes a Patient Representative nominated by this PPI Volunteer group.

### Dissemination of research to the diabetes community

In communicating the study results to as wide as possible an audience, special effort will be made to reach patients with type 2 diabetes, and people at risk of developing type 2 diabetes. One of the aims of the research is to raise greater awareness of the cardiovascular complications of diabetes and of possible preventative measures. We will take part in the Diabetes Wellness Days (drwf.org.uk/get-involved/diabetes-wellness-network), which bring together under one roof a wealth of information for people living with diabetes, their family members, carers, friends and those with an interest in the condition. These events will also provide information on the importance of supporting and taking part in research in this field. Work published in peer-reviewed journals will be shared with the press offices of the University where appropriate, whilst ensuring that results are presented to the media in an appropriate and accurate manner. Alongside press releases, we will pitch articles to The Conversation (theconversation.com/uk/health) – an online news website that partner’s researchers with journalists to write about topical news stories – to explain our findings in greater depth, to a large, non-specialist, audience.

Finally, each participant will be given detailed feedback on their cardiac function and their glycaemic control respectively at the end of the study. The primary care physicians will receive a letter stating which drug had a better impact on improvement in glycaemic control after completion of the trial once the results are unblinded.

We are most grateful to the members of Leeds PPI group and Leeds Diabetes UK Volunteer Group for their contributions to design and recruitment process of this study.

### Objectives and outcome measures

#### Primary objective

To explore and compare the impact of Empagliflozin and Sitagliptin to modulate myocardial perfusion.

#### Secondary objectives

To explore and compare the impact of Empagliflozin and Sitagliptin on: **(**i) Myocardial fibrosis; **(**ii) myocardial function; (iii) aortic distensibility; (iv) plasma glucose variability (time in range, hypoglycaemic exposure and glycaemic variability).

#### Primary outcome measure

Change in myocardial perfusion reserve in the remote territory.

#### Secondary outcome measures

After treatment change in:( i) Myocardial extra cellular volume fraction (ECV); (ii) myocardial perfusion reserve in the infarcted territory; (iii) left ventricular ejection fraction, (iv) right ventricular ejection fraction; (v) left ventricular strain (peak circumferential systolic strain and peak early diastolic strain rate, and global longitudinal strain); (vi) aortic distensibility; (vii) Libre Pro FreeStyle Sensor measured parameters (TIR, hypoglycaemic exposure and glycaemic variability).

### Methods and analysis

#### Study Population

Twenty-eight patients with underlying T2D and ischaemic heart disease will be recruited.

### Inclusion and exclusion criteria

#### Recruitment and data collection

Patients will be recruited from the Leeds Teaching Hospitals NHS Trust (LTHT) or from GP practices. Participants will be identified from cardiology wards, cardiology clinics and the cardiac rehabilitation clinics in LTHT. Suitable participants will be approached by a member of the clinical team and given a patient information sheet (PIS) if interested. Recruitment of T2D patients with no history of recent hospital admission is supported by the National Institute for Health Research Clinical Research Network (NIHR-CRN). NIHR-CRN team will approach GP surgeries with study information and practices which expressed interest in helping with recruitment to the CRN will become ‘patient identification centres’ (Table 1).

**Table 1.** Inclusion and exclusion criteria.

Inclusion criteria	Exclusion criteria
• Diagnosis of T2D • Currently on Metformin as a single or dual therapy • HbA1c > 48 mmol/mol (>58 mmol/mol if on a Sulphonylurea) within 3 months of recruitment • Age between 18 and 84 years old • Ability to provide informed consent • Prior diagnosis of ischaemic heart disease by angiography or a positive non-invasive stress test for ischaemia	• History of CABG or need for further revascularisation • History of type 1 diabetes or previous diabetic ketoacidosis • Current treatment with Sitagliption or Empaglifozin • Any absolute contraindication to CMR • Contraindication to adenosine • Severe asthma • Known allergy to contrast medium (gadolinium) • Renal dysfunction (eGFR < 60) • Pregnancy or breast feeding

CABG: coronary artery bypass grafting; CMR: cardiac magnetic resonance; eGFR: estimated glomerular filtration rate; T2D: type 2 diabetes.

## Study investigations

**Figure 2.**
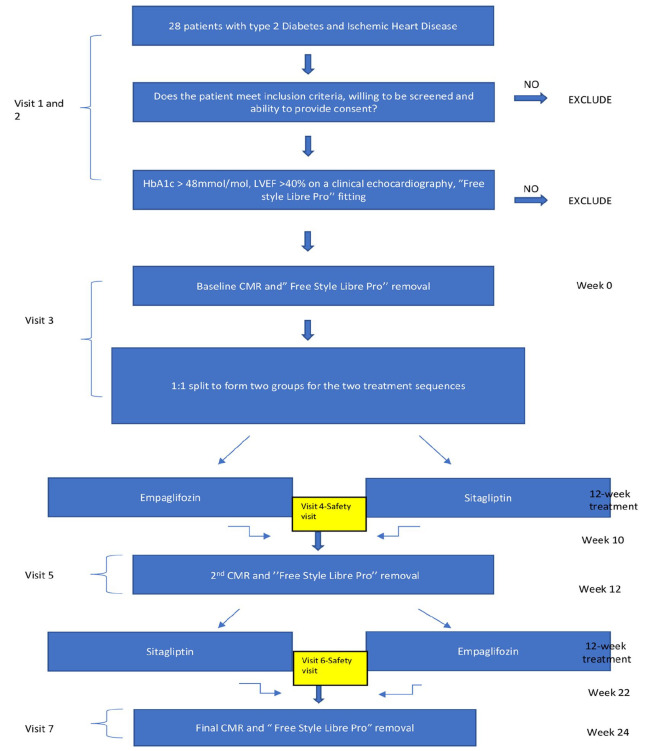
Study flow chart. Screening (Visits 1 and 2): Screening assessments will be performed over two visits and will include a review of medical history and concomitant medications, and a review of history of diabetes and complications. Screening tests will include blood tests for fasting serum glucose, insulin, full blood count (FBC), urea and electrolytes (U&E), glycated haemoglobin (HbA1c). The most recent clinical echocardiography report will be reviewed, and the Libre Pro Sensor fitting will be performed at this visit. Visit 3: First CMR assessments will be scheduled for the third study visit to the research centre (at least 2 months after myocardial infarction, revascularisation procedure or unstable angina episode). Prior to CMR imaging the Libre Pro Sensor will be removed and randomisation to one of the two medications will be undertaken. Visit 4: Blood tests including fasting serum glucose, insulin, FBC, U&E and HbA1c will be performed and a second Libre Pro Sensor will be fitted. Visit 5: The Libre Pro Sensor will be removed and the second CMR scan will be undertaken. Visit 6: Further blood tests including fasting serum glucose, insulin, FBC, U&E and HbA1 will be performed and the final Libre Pro Sensor will be fitted. Visit 7 (Final Visit): The Libre Pro Sensor will be removed and the final CMR scan will be performed.

Study flow chart, details of all study visits with assigned investigations and analysis techniques are provided in Figure 2. The assessments listed below will be carried out at each visit in the Advanced Imaging Centre at the Leeds General Infirmary.

### Informed consent

Written informed consent will be taken from all patients.

### Blood tests

Fasting bloods including glucose, serum insulin, C peptide, glycated haemoglobin (HbA1c), urea and electrolytes, kidney function tests and full blood count will be obtained.

### FreeStyle Libre Pro Sensor

Glucose profile in the study participants will be studied in detail using FreeStyle Libre Pro Sensor. The FreeStyle Libre Pro Sensor is a blinded sensor worn for 14 days which takes a glucose measurement every 15 min (96 readings/day = total of 1344 glucose readings over 2 weeks). The FreeStyle Libre Pro Sensor allows measuring: (i) time in range (TIR): time spent between 3.9 and 10.0 mmol/L/day (clinical target > 70% in this range),^39^ (ii) hypoglycaemic exposure: analysed at two levels as time spent at <3.9 mmol/L (target < 4%) and <3.0 mmol/L (target < 1%), in accordance with international guidelines^39^ and (iii) glycaemic variability (GV): assessed as coefficient of variation (CoV, target < 36%).

### Cardiovascular magnetic resonance

**Figure 3.**
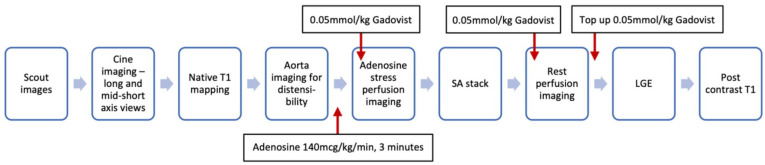
CMR protocol. LGE: late gadolinium enhancement; LV: left ventricular; RV: right ventricular; SA: short axis.

Patients will undergo CMR studies at 3.0 Tesla (Magnetom Prisma, Siemens, Germany) for determining structural, functional and ischaemic changes in the heart (biventricular size, function, strain, aortic distensibility, myocardial perfusion, fibrosis and scarring). The scan will be repeated using the same CMR study protocol (Figure 3) after each treatment allocation. Cardiac imaging receiver coil configuration will be used, and electrocardiogram (ECG) gating will be performed.

CMR will include scout and cine imaging to assess LV volumes, mass and ejection fraction, myocardial strain parameters. For aortic distensibility measurements, high temporal resolution multi-phase SSFP cine imaging will be acquired transverse to the ascending and descending thoracic aorta at the level of the pulmonary artery bifurcation. Aortic pulse wave velocity will then be assessed using identical geometric planning with retrospectively gated, through-plane, phase-contrast velocity encoded images. Adenosine (140 μg/kg/min) will then be infused for at least 3 min. Subsequently, gadolinium-based contrast (Gadovist^®^, Bayer Pharma, Berlin, Germany) will be injected for first-pass perfusion imaging.^40^ If the haemodynamic response was inadequate (HR increase < 10 bpm or systolic blood pressure decrease < 10 mmHg) then the infusion rate will be increased up to a maximum of 210 mcg/kg/min (maximal infusion duration 7 min). Adenosine will then be discontinued and, after at least 20 min to allow washout, another bolus of gadolinium (0.05 mmol/kg) will be given for rest perfusion imaging. Data acquisition will use a multi-slice, free-breathing, saturation recovery pulse sequence with fast low angle shot (FLASH) readout, acquired over 60 heartbeats. Late gadolinium enhancement (LGE) in matching LV short- and long-axis planes will be carried out more than 8 min after rest perfusion imaging. Postcontrast T1 mapping will then be carried out 15 min following the last contrast injection (Table 2).

**Table 2.** CMR pulse sequence.

Major components	LV, RV, LA size and function	Late gadolinium enhancement	T1-mapping	Perfusion
Preferred pulse sequence	Fast gradient echo sequence; 10–12 slices; 30 phases;10//0 mm (free breathing with MOCO or breath holds of 5 s)	PSIR MOCO SSFP (if not available, then PSIR without MOCO)	Native T1 mapping; 5s3s MOLLI; three slices (breath hold 11 s)	Kellman pixel-wise perfusion mapping
			Post contrast T1; 4s3s2s MOLLI (breath hold 12 s)	T1 weighted saturation recovery prepared gradient echo sequence in 3–4 short axis slices; free breathing with MOCO

LGE: late gadolinium enhancement; LV: left ventricular; RV: right ventricular; SA: short axis; LA: left atrium; PSIR: phase sensitive inversion recovery; MOCO: motion corrected; SSFP: steady-state free precession.

### Cardiovascular Magnetic Resonance analysis

All imaging will be analysed blinded to patient details. CMR data will be assessed quantitatively using commercially available software (CVI42, Circle Cardiovascular Imaging Inc, Calgary, Canada). Epicardial and endocardial borders will be traced offline on the LV cine stack at end-diastole and end-systole to calculate end-diastolic and end-systolic LV volumes, stroke volume, ejection fraction and LV mass, as previously described.^41^ Tissue tracking analysis will be performed using CVI42. The peak systolic circumferential strain, global longitudinal strain and diastolic strain rate data will be measured. Myocardial perfusion (MP) in mL/min/g will be assessed using in-line motion corrected perfusion mapping implemented within the Gadgetron software framework as described in Kellman et al.^40^ Global (average myocardial perfusion for 16 segments) and segmental MP will be measured. MP reserve (MPR) will also be calculated defined as the ratio between MP at stress over rest. Focal and diffuse fibrosis will be assessed using LGE and native and post contrast T1 to estimate the myocardial ECV.^42^ Aortic dimensions and aortic distensibility will be calculated by standard techniques.^43^

### Randomisation

Eligible patients will be randomised in a 1:1 ratio for the order in which they will receive Empagliflozin 10 mg once daily and Sitagliptin 100 mg once daily, respectively. Participants will undergo randomisation at the baseline visit, using the randomisation schedule generated by the LTHT Clinical Trials team. Randomisations will be achieved using minimisation incorporating a random element, via a computer-generated program, that will allocate patients in a 1:1 ratio after taking account of age, gender and BMI. Participants will be given labelled medication so they will be aware of which medication they are taking on each occasion. Instructions on how and when to take the prescribed medication in its licensed dose will be issued at the time of enrolment by the study investigators who are medically qualified and General Medical Council registered. There will be no washout period during this study which is recognised as a limitation of the study.

### Monitoring

Recommended monitoring for both Empagliflozin and Sitagliptin is to check renal function prior to initiation and periodically throughout administration – stated as at least yearly for Empagliflozin and periodically for Sitagliptin. Accordingly, renal function tests will be tested prior to initiating medication and prior to each CMR scan; each visit, therefore prior to initiation and after 10 weeks of treatment (2–3 weeks before each scan). All participants will be fully informed about these potential side effects.

### Statistics

#### Power calculations

With 28 participants, the study will have 80% power (binomial test) to show a change of 12% in myocardial blood flow.^44^

#### Statistical analysis

Outcome measures at follow-up will be compared between groups using linear regression models, adjusted for baseline measures of the outcome. Differences between randomised groups will be tested overall, and then pairwise differences between groups will be estimated separately.

#### Planned subgroup analyses

Interaction tests within regression models will be used to formally test for differences in treatment effects between subgroups defined by age and sex, as secondary analyses. Any other subgroup analyses will be carried out in an exploratory, hypothesis-generating manner.

#### Compliance and loss to follow up

The participants will be free to withdraw from the study at any time. Where patients wish to withdraw from the study, clarification of the extent of withdrawal will be sought and documented in the case report form. Participants who withdraw from the study will not be replaced.

#### Potential risks and hazards

The safety of the treatment will be evaluated through collection of adverse events, with a particular focus on adverse events relevant to Empaglifozin and Sitagliptin. At the study completion/early termination visit, each patient will be instructed to report any subsequent adverse events that the patient’s GP believes could be related to the study drug treatment or study procedures. The patient information leaflets will include all adverse events considered relevant to treatment with Empaglifozin and Sitagliptin and to MR studies.

### Identifying and reporting adverse events

Participants will be instructed to contact the investigators if any untoward events occur during the clinical study. This includes any unintentional, unfavourable clinical signs or symptoms, any new illnesses or disease, or deterioration of existing disease, and any clinically relevant deterioration in any laboratory assessments of clinical tests. All non-serious or expected adverse events will be recorded on the study CRF and will be reported to the main Research and Ethics Committee (REC).

In keeping with National Health Service Health Research Authority (HRA) guidelines, reports of Serious Adverse Events (SAEs) or Serious Adverse Reactions (SARs) that are related to the study and unexpected will be reported to the sponsor within one working day of the research team becoming aware of the event, and submitted to the REC using the HRA Non-CTIMP safety report to REC form within 15 days of the chief investigator becoming aware of the event. Events will be followed up until the event has resolved or an outcome has been reached.

## Discussion

Given recent findings on efficacy and benefits, SGLT2 inhibitors have rapidly established a significant role in the treatment of diabetes. In the EMPAREG OUTCOME Trial, in T2D patients both with and without a history of HF, Empagliflozin reduced hospitalisation for HF with almost immediate beneficial effect. SGLT2 inhibitors also cause a modest rapid reduction in weight, haemoconcentration and reduced blood pressure, consistent with a diuretic effect, which could improve congestion.^2,4,5^ Though SGLT2 inhibitors were initially developed to target hyperglycemia, given these beneficial effects reducing the risk of HF hospitalisations, SGLT2 inhibitors began to emerge as potential drugs to prevent HF in populations with and without diabetes.^2,4,5^ While the mechanisms for the beneficial CV effects of SGLT2 inhibitors in T2D patients remain to be elucidated, the postulated mechanisms include: (i) improvement in ventricular loading conditions through a reduction in preload with natriuresis and osmotic diuresis,^45,46^ and afterload with reduction in BP and improvement in vascular function^35,47^; (ii) restoration of cellular energy homeostasis by activation of AMP-activated protein kinase, inducing mild ketosis and inducing a metabolic switch toward utilisation of ketone bodies, fatty acids and branch chain amino acids^48,49^; (iii) reduction of cardiac fibrosis; (iv) alteration in adipokines, cytokine production and epicardial adipose tissue mass; (v) significant reduction in LV mass.^50^ Table 3 summarises the details of recent clinical studies exploring the mechanisms for the beneficial CV effects of Empagliflozin. However, until definite answers are given, it appears that SGLT2 inhibitors exert their favourable HF outcome by pleiotropic mechanisms beyond the reduction of glucotoxicity and diuresis. This study aims to increase our understanding of the fundamental benefits of SGLT2 inhibition which could inspire a change in practice in the future either as an adjunct or as a first-line treatment for diabetes.

**Table 3.** Clinical studies exploring the mechanisms for the beneficial CV effects of Empagliflozin.

Study	Imaging modality	Journal/year	Recruited cohort	Primary objective	Results
Verma et al.^51^	TTE before and 3 months after	*Diabetes Care* 2016	10 people with T2D and CVD	Change in LVSF and LV mass index	Improved LV diastolic function according to early lateral e′
					Reduced LV mass index
					No difference in LV volumes and LV EF
Sakai et al.^52^	TTE before and 3 months after	*Circulation* 2019	184 people withT2D and HFpEF	Assess improvement in vascular function and vascular structure in patients with HFpEF	Improved LV diastolic function according to the E/A and E/e′ ratio
Effects of Empagliflozin treatment on cardiac function and structure in patients with type 2 diabetes: A cardiac magnetic resonance study Cohen et al.^53^	Cardiac MRI before and 6 months after	*Internal Medicine Journal* 2019	25 people withT2D (17 drug and 8 placebo)	Assess cardiac functional and structural changes based on CMR measurements	Reduced LV end-diastolic volume
					No difference in LV mass, LV EF, atrial volumes and markers of cardiac fibrosis
EMPA heart Cardio-link six trial Verma et al.^50^	Cardiac MRI before and 6 months after	*Circulation* 2019	97 people withT2D and CVD (49 drug and 48 placebo)	Six-month change in LV mass indexed to body surface area from baseline as measured by cardiac magnetic resonance imaging	Significant reduction in LV mass indexed to body surface area after 6 months
					No difference in LV EF and LV end-systolic volume
Are the ‘Cardiac Benefits’ of Empagliflozin Independent of Its Hypoglycaemic Activity? (ATRU-4 EMPA-TROPISM) Santos-Gallego et al.^54^	Cardiac MRI before and 6 months after	*JACC* 2020	80 people withT2D and HFrEF	Change in LV end-systolic and end-diastolic volumes Change in LV EF	Improvement in LV volumes, LV mass, LV systolic function, functional capacity and quality of life when compared with placebo
Effect of Empagliflozin on left ventricular volumes in patients with type 2 diabetes, or prediabetes, and heart failure with reduced ejection fraction (SUGAR-DM-HF) Lee et al.^55^	Cardiac MRI before and 36 weeks after	*Circulation* 2020	105 patients with NYHA functional class II to IV with a left ventricular (LV) ejection fraction ⩽40% and type 2 diabetes or prediabetes	Changes from baseline to 36 weeks in LVESVi and LV GLS measured using cMRI	Reduction in LV volumes in patients with HFrEF and type 2 diabetes or prediabetes

A: mitral peak A-wave velocity; CVD: cardiovascular disease; E: mitral peak E-wave velocity; e′: early annular tissue Doppler velocity; EF: ejection fraction; HFpEF: heart failure with preserved ejection fraction; HFrEF: heart failure with reduced ejection fraction; LV: left ventricular; MRI: magnetic resonance imaging; SGLT2: sodium-glucose cotransporter 2; T2D: type 2 diabetes; TTE: transthoracic echocardiography; cMRI: cardiac magnetic resonance imaging; LVSF: left ventricular systolic function; LV GLS: LV global longitudinal strain; LVESVI: LV end-systolic volume indexed to body surface area.
